# Positive and Negative Changes in the Electrical Conductance Related to Hybrid Filler Distribution Gradient in Composite Flexible Thermoelectric Films Subjected to Bending

**DOI:** 10.3390/nano13071212

**Published:** 2023-03-29

**Authors:** Lasma Bugovecka, Krisjanis Buks, Jana Andzane, Annija Dinija Miezubrale, Juris Bitenieks, Janis Zicans, Donats Erts

**Affiliations:** 1 Institute of Chemical Physics, University of Latvia, Jelgavas str. 1, LV-1004 Riga, Latvia; 2 3D Strong Ltd., Instituta Str. 36-17, LV-2130 Ulbroka, Latvia; 3 Institute of Polymer Materials, Riga Technical University, 3/7 Paula Valdena Street, LV-1048 Riga, Latvia; 4 Faculty of Chemistry, University of Latvia, Raina Blvd 19, LV-1586 Riga, Latvia

**Keywords:** flexible composite thermoelectric film, hybrid filler distribution gradient, antimony telluride-multiwalled carbon nanotubes heterostructures, thin film bending, electrical conductance change, thin film compression and tension

## Abstract

P-type multiwalled carbon nanotubes (MWCNTs), as well as heterostructures fabricated by direct deposition of inorganic thermoelectric materials as antimony and bismuth chalcogenides on MWCNT networks are known as perspective materials for application in flexible thermoelectric polymer-based composites. In this work, the electrical response of three types of Sb_2_Te_3_-MWCNT heterostructures-based flexible films—free standing on a flexible substrate, encapsulated in polydimethylsiloxane (PDMS), and mixed in polyvinyl alcohol (PVA) is studied in comparison with the flexible films prepared by the same methods using bare MWCNTs. The electrical conductance of these films when each side of it was subsequently subjected to compressive and tensile stress during the film bending down to a 3 mm radius is investigated in relation to the distribution gradient of Sb_2_Te_3_-MWCNT heterostructures or bare MWCNTs within the film. It is found that all investigated Sb_2_Te_3_-MWCNT films exhibit a reversible increase in the conductance in response to the compressive stress of the film side with the highest filler concentration and its decrease in response to the tensile stress. In contrast, free-standing and encapsulated bare MWCNT networks with uniform distribution of nanotubes showed a decrease in the conductance irrelevant to the bending direction. In turn, the samples with the gradient distribution of the MWCNTs, prepared by mixing the MWCNTs with PVA, revealed behavior that is similar to the Sb_2_Te_3_-MWCNT heterostructures-based films. The analysis of the processes impacting the changes in the conductance of the Sb_2_Te_3_-MWCNT heterostructures and bare MWCNTs is performed. The proposed in this work bending method can be applied for the control of the uniformity of distribution of components in heterostructures and fillers in polymer-based composites.

## 1. Introduction

Global demand for energy is ever-increasing, which accelerates the consumption of non-renewable resources. To reduce resource consumption and to develop an energy-efficient society, energy utilizing processes should be optimized, as about two-thirds of primary energy are wasted as heat [1]. Around 60% of waste heat lost is of low or ultralow grade (heat at temperatures between ambient temperature and 80 °C) [2]. While a perspective method for transforming waste thermal energy back into useful electrical power is to use effective thermoelectric generators (TEGs). However, conventional commercially available TEGs are made of brittle inorganic thermoelectric (TE) materials and have a solid rectangular shape, which limits their usability for the low- and ultralow-grade waste heat capturing as these heat losses mostly occur from curved heated surfaces as, for example, hot pipes or human body. Thus, capturing and conversion of low- and ultralow-grade waste heat requires the development of flexible TE materials.

A perspective type of flexible TE materials for low- and ultra-low-grade waste heat conversion to electricity are nanocomposites consisting of a polymer matrix filled with a TE filler as carbon nanotubes (CNT) [3] or nanostructured inorganic materials for near-room temperature applications (Bi_2_Se_3_, Bi_2_Te_3_, Sb_2_Te_3_) [4,5,6,7]. Among the polymer matrices, insulating polymers are considered as more environmentally-friendly, stable, and more affordable in comparison with their electrically-conductive counterparts [8,9]. Recently, it was shown that using CNTs as a scaffold for the deposition of inorganic thermoelectric nanostructures as Bi_2_Te_3_, Bi_2_Se_3_ or Sb_2_Te_3_ allows to obtain effective flexible thermoelectric material [10,11,12]. Such networks of Bi_2_Se_3_-CNT and Sb_2_Te_3_-CNT heterostructures can also be used for the fabrication of effective flexible thermoelectric composites based on polyvinyl alcohol (PVA) matrix [6,7,13]. For the fabrication of these composites, the TE fillers were prepared by direct deposition of inorganic Bi_2_Se_3_ or Sb_2_Te_3_ nanostructures on a multiwalled CNT network [12], and further mixed-in or encapsulated-by a polymer [6,7,13]. Both mixing and encapsulation approaches resulted in a gradient distribution of the TE filler across the polymer matrix due to the fabrication issues. In the mixing approach, the filler may partially settle on the bottom side of the polymer matrix due to the impact of gravity during the drying of the polymer. However, despite the potential influence of the filler distribution gradient on the properties of the composite, this issue is usually not given sufficient attention. In turn, the encapsulation technology implies location of the CNT-TE filler along one side of the composite film [7]. For the reliable application of flexible TE composites, it is crucial to understand the impact of uneven distribution of filler in flexible TE materials on their properties upon bending. When bending films, their outer and inner surfaces are always subjected to tension and compression stress respectively. However, quite a limited amount of research attention is paid to the study of the resistance response of the electrically conductive thin films to both compression and tension during the bending. Up to date, most of such studies were devoted to the durability of silver paint electrodes deposited on polymer substrates for wearable electronics [14,15], silver networks covered by polymer for flexible thin film heaters [16], and flexible metal oxide electrodes on polymer substrates for touch-screen panels [17] and flexible OLED applications [18]. In most of these works, the resistance measurements were applied to determine the limits for mechanical bending and durability. Some of the works report the difference between the electrical conductance change (commonly increase from the value of not bent sample) in respect to the bending direction (inner/outer), when approaching critical bending radius [14,18]. However, almost no attention is paid to the analysis of mechanisms underlying these changes in the electrical conductance of the samples. In addition, generally such studies do not take into account that during the bending, outer and inner surfaces of the film are subjected simultaneously to the opposite (tensile/compressive) types of bending stress. In the case of uneven distribution of the nanostructured functional filler within the film, the changes in properties on the sides of the sample subjected to tensile and compressive stress significantly differ, which will be reflected in the electrical conductance changes. To the best of our knowledge, up to date there is no reports on a systematic and detailed study devoted to the electrical response of thermoelectrical composite materials, based on insulating polymer matrix combined with the functional nanostructured filler, in respect to the impact of filler distribution gradient on the conductance changes when subjected to compressive/tensile stress.

In this work, a systematic study of the electrical conductance changes during the bending of three different types of Sb_2_Te_3_-MWCNT heterostructures and bare MWCNT-based thermoelectrical flexible films—the free-standing on a flexible polymer substrate, encapsulated in polydimethylsiloxane (PDMS), and mixed in PVA–is presented. The impact of compression and tension during the bending on the changes in the sample conductance are studied in detail in respect to the processes occurring in the electrical contacts between the MWCNTs and between the components of Sb_2_Te_3_-MWCNT heterostructures. It was found that the differences in the behavior of different types of Sb_2_Te_3_-MWCNTs heterostructures-based composites when subjected to compression and tension are related to the impact of gradient distribution of Sb_2_Te_3_ nanostructures. Similar impact of gradient distribution of bare MWCNTs on the conductance changes upon bending was observed for the samples prepared by mixing MWCNTs with PVA.

## 2. Materials and Methods

MWCNTs were synthesized via chemical vapor deposition method in a quartz tube reactor (3D Strong Ltd., Ulbroka, Latvia). The process occurred at 850 °C and at carrier gas (Ar) gas flow rate 20 mm/s for 20 min. A mixture on benzene (C_6_H_6_, CAS:71-43-2, 99.8% Sigma-Aldrich, Inc., St. Louis, MI, USA), and pyridine (C_5_H_5_N, CAS:110-86-1, 99.8%, Sigma-Aldrich, Inc., St. Louis, MI, USA) in 3:1 by volume, with dissolved in this solution 2 wt% of ferrocene (Fe(C_5_H_5_)_2_, CAS:102-54-5, 98%, Sigma-Aldrich, Inc., St. Louis, MI, USA) was used for the formation of catalytic particles for the MWCNT growth.

MWCNT networks were prepared by spray-coating technique on glass and polyimide film (DuPont™ Kapton^®^, Wilmington, DE, USA) substrates. Polyimide film is flexible, with good chemical and thermal stability from −269 to +300 °C, which makes it suitable substrate for physical vapor deposition process.

Hybrid Sb_2_Te_3_-MWCNT networks were synthesized using a catalyst-free physical vapor deposition method described in [12,19,20] by direct deposition of Sb_2_Te_3_ (99.999%, CAS: 1327-50-0, Alfa Aesar, Kandel, Germany) on MWCNT networks in a quartz tube single zone vacuum furnace (OTF-1200X, MTI Corp., Richmond, CA, USA). By varying thickness of MWCNT networks and the time of synthesis, the desired weight percent (wt%) of MWCNT’s in the hybrid structures were achieved (the samples were weighed before each step using analytical scales (KERN ABP 200-5DM, max 220 g, ±1 mg, Balingen, Baden-Wuerttemberg, Germany). For the fabrication of flexible films containing encapsulated Sb_2_Te_3_-MWCNT or bare MWCNTs, the heterostructures were deposited on polyimide substrates, then coated with PDMS (Sigma-Aldrich, Inc., St. Louis, MI, USA), cured for 10 min in 150 °C, and then the resulting elastic film was lifted off the glass using tweezers. For the fabrication of mixed composite, the Sb_2_Te_3_-MWCNT heterostructures were deposited on glass substrates, then scrapped off the substrates and mixed in PVA (Sigma-Aldrich, Inc., St. Louis, MI, USA) as described elsewhere [13].

Morphological and structural characterization of the samples was performed by field-emission scanning electron microscope (SEM) (S-4800, Hitachi, Tokyo, Japan) equipped with energy-dispersive X-ray diffraction analyser (EDX) (Bruker XFlash Quad 5040, Billerica, MA, USA), transmission electron microscope (FET Technai GF 20, FEI Company, Hillsboro, OR, USA), and X-ray diffractometer (XRD, Bruker D8 Discover, Billerica, MA, USA). Identification of the diffraction peaks was performed using the ICDD database PDF-2/Release 2021 (Ref. cards PDF 01-085-4141 (Sb_2_Te_3_), PDF 00-001-0727 (Te), and PDF 01-075-1565 Sb_2_O_3_).

2-point bending experiments were carried out with a home-build device, allowing simultaneous bending of the sample and measuring of its electrical resistance using Keithley 6430 Sub-Femtoamp remote source meter (Keithley Instruments, Cleveland, OH, USA) combined with a custom software). The resistance of the samples during bending was measured at a constant voltage of 0.1 V applied to the sample.

## 3. Results and Discussion

### 3.1. MWCNT-Sb_2_Te_3_ Network Distribution in Inert Polymer Matrix

**Figure 1:**
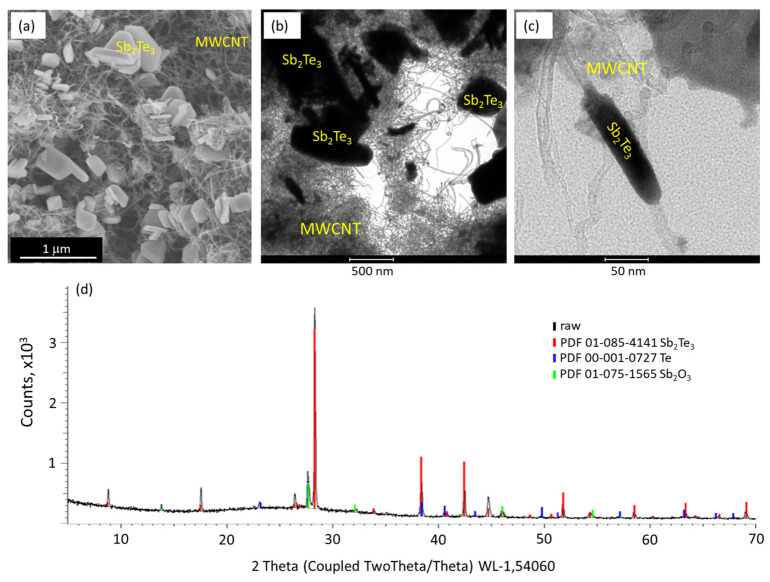
Scanning (**a**) and transmission electron microscope (**b**,**c**) images of Sb_2_Te_3_ nanostructures grown on a MWCNT network via physical vapor deposition, illustrating formation of Sb_2_Te_3_ clusters on the MWCNT network; (**d**) X-ray diffraction pattern of the deposited Sb_2_Te_3_ nanostructures.

Spray-coating of the MWCNTs on the substrates results in formation of ~2–2.5 μm thick networks with the uniform distribution of the MWCNTs throughout the network. During further physical vapor deposition of Sb_2_Te_3_, it forms clusters rather than uniform coating on MWCNTs (Figure 1a–c).

Such deposition is consistent with the previous reports on the growth of Sb_2_Te_3_ nanostructures on MWCNT surfaces and is related to the deposition mechanism [12]. The XRD pattern of the deposited Sb_2_Te_3_ nanostructures (Figure 1d) showed the presence of peaks characteristic for Sb_2_Te_3_ in a powder form (Figure 1d, red curve), indicating random orientation of the Sb_2_Te_3_ nanostructures. In addition, low-intensity diffraction peaks related to Sb_2_O_3_ (Figure 1d, blue curve) and Te (Figure 1d, green curve) were observed, indicating the presence of native oxide layer on the Sb_2_Te_3_ nanostructures, as well as possible inclusion of clusters of Te in the Sb_2_Te_3_-MWCNT heterostructures. These results are consistent with the previously reported for the MWCNT-Sb_2_Te_3_ heterostructures [13].

**Figure 2:**
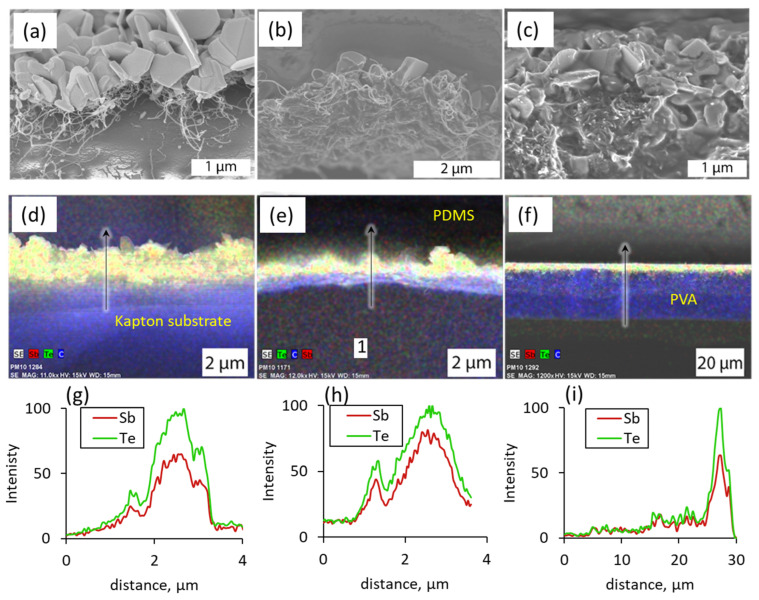
SEM images (**a**–**c**), EDS mapping (**d**–**f**) and EDS elemental line-scan profile (**g**–**i**) of the cross section of flexible films containing Sb_2_Te_3_-MWCNT heterostructures: (**a**,**d**,**g**) free standing on polyimide surface; (**b**,**e**,**h**) encapsulated in PDMS; and (**c**,**f**,**i**) mixed in PVA. Arrows in (**d**–**f**) represent the scanning direction.

The initial formation of Sb_2_Te_3_ clusters within the top layer of 2–2.5 μm thick MWCNT network prevents its further penetration by Sb and Te ions and thus results in a gradient distribution of the Sb_2_Te_3_ nanostructures across the MWCNT network as it can be seen in Figure 2a, illustrating free-standing Sb_2_Te_3_-MWCNT heterostructures, formed by physical vapor deposition of Sb_2_Te_3_ on prefabricated on a flexible polyimide substrate MWCNT networks. The total thickness of the sample consisting of free standing on the polyimide substrate Sb_2_Te_3_-MWCNT heterostructures was ~20 μm (Figure 2a), where ~16.5–17 μm is the thickness of the polyimide substrate and ~3–3.5 μm is the thickness of Sb_2_Te_3_-MWCNT heterostructures (Figure 2d). The cross-sectional distribution of the Sb_2_Te_3_ and MWCNT components in these heterostructures showed that the concentration of Sb and Te on the top of MWCNT networks is higher in comparison with the bottom (Figure 2d,g) as initial growth of Sb_2_Te_3_ nanostructures on the top layer of MWCNT network complicates penetration of material vapors to the inner layers of MWCNT network. In the free-standing Sb_2_Te_3_-MWCNT heterostructures, the highest concentration of Sb_2_Te_3_ was observed in a 1–1.5 μm thick top layer (Figure 2d,g).

Encapsulation of free standing MWCNT networks and Sb_2_Te_3_-MWCNT heterostructures in PDMS (Figure 2b) with following lift off from the substrate resulted in obtaining of thick films, consisting of the layer of encapsulated MWCNTs or heterostructures coated with the ~ 500 μm thick layer of bare PDMS, which determines the total thickness of these films. Encapsulation practically did not affect the distribution of elements in the MWCNT networks or Sb_2_Te_3_-MWCNT heterostructures in comparison with the free-standing sample, thus, the thickness of the encapsulated MWCNT and Sb_2_Te_3_-MWCNT layer remained within 2–2.5 μm and 3–3.5 μm respectively, with highest concentration of Sb_2_Te_3_ within 1–1.5 μm (Figure 2e,h).

In turn, the samples where the Sb_2_Te_3_-MWCNT heterostructures or bare MWCNTs were mixed in PVA had total thickness of ~30 μm (Figure 2c). The distribution gradient of the Sb_2_Te_3_-MWCNT in the film detected by the EDX (Figure 2f,i) showed that the concentration of Sb_2_Te_3_ on one side of the film is up to 10 times higher than on the opposite side. The thickness of the layer with highest Sb_2_Te_3_ concentration is approximately 5 µm (Figure 2i). Presuming that observed distribution gradient of Sb_2_Te_3_ represents the location density of Sb_2_Te_3_-MWCNT heterostructures in the mixed films (as bare MWCNTs cannot be distinguished by the EDX technique from the carbon-based PVA), the difference in Sb_2_Te_3_ concentrations between the top and bottom surfaces of the film can be explained by partial settling of the Sb_2_Te_3_-MWCNT heterostructures on the bottom surface of the PVA film during its curing. Similar effect of partial settling of filler was detected for bare MWCNTs-based samples. It was observed that the top and bottom surfaces of the film prepared by mixing MWCNTs with PVA had dark and grey colors respectively, indicating higher concentration of the MWCNTs at the bottom side of the film. The presence of the MWCNTs throughout the PVA matrix and their uneven distribution was confirmed by the measurements of the electrical resistance of top and bottom surfaces of the film. Both surfaces were electrically conductive, which indicates the presence of MWCNTs. However, the electrical resistance of the top (grey) surface of the sample was ~1.7 times higher in comparison to the bottom (dark) surface (6.6 kΩ vs. 3.9 kΩ). This confirms different concentrations of MWCNTs near the top and bottom surfaces of the sample.

### 3.2. Electrical Conductance of Sb_2_Te_3_-MWCNT Heterostructures and Bare MWCNTs -Based Flexible Films with Gradient and Uniform Component Distribution under Compression and Tension

**Figure 3:**
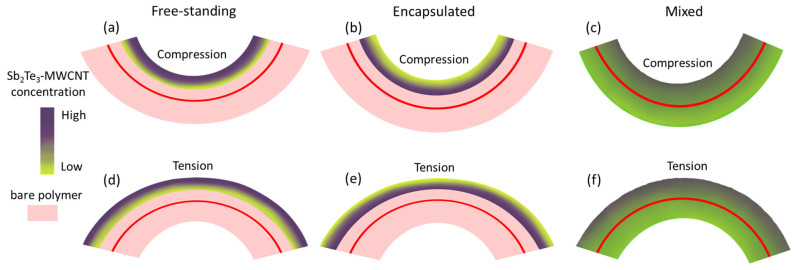
A schematic representation of flexible films with unevenly distributed Sb_2_Te_3_-MWCNT heterostructures, bending in compression or tension mode relatively to the side with the highest concentration of Sb_2_Te_3_: (**a**,**d**) free-standing on a polyimide substrate; (**b**,**e**) encapsulated in PDMS; and (**c**,**f**) mixed with PVA The red line represents the middle line of the sample.

While bending a film, there is a compressive stress on its inner side and a tensile stress on the outer side. Bending of the films to both directions allowed to determine the response of electrical conductance of the layer of Sb_2_Te_3_-MWCNT heterostructures on both compression and tension. In this work, the thin film side with the highest Sb_2_Te_3_ concentration (also MWCNT for mixed in polymer) is used as reference for bending mode. “Compression” denotes that the film is bent so that the side with the highest Sb_2_Te_3_ (MWCNT) concentration is subjected to the compressive stress, and “tension” denotes that the film is bent so that the side with the highest Sb_2_Te_3_ concentration is subjected to tensile stress (Figure 3a,b,d,e; red line represents the middle line of the sample). For the free-standing and encapsulated bare MWCNTs-based samples it is presumed that the distribution of the MWCNTs in the network is uniform. “Compression” denotes that the film is bent so that the MWCNT network is subjected to compressive stress, and “tension” denoted that this network is subjected to tensile stress. For the films, prepared by mixing the Sb_2_Te_3_-MWCNT heterostructures or bare MWCNTs with PVA, where the distribution of MWCNT is gradient, “compression” denotes that the side of the film with the higher Sb_2_Te_3_-MWCNT heterostructures or bare MWCNTs concentration is subjected to compressive stress, and “tension” denotes that this side of the film is subjected to the tensile stress during the bending (Figure 3c,f).

It should be noted that in case of free-standing and encapsulated Sb_2_Te_3_-MWCNT or bare MWCNTs, during the bending the whole layer is subjected to compression or tension relative to the middle line of the sample (Figure 3a,b,d,e). In contrast, in case of Sb_2_Te_3_-MWCNT heterostructures or bare MWCNTs mixed with PVA, while one side of the sample (for example, with higher concentration of heterostructures or MWCNTs) is subjected to compression, the other side of the sample (with lower concentration of heterostructures or MWCNTs) is subjected to tension relative to the middle line of the sample, and vice versa (Figure 3c,f).

**Figure 4:**
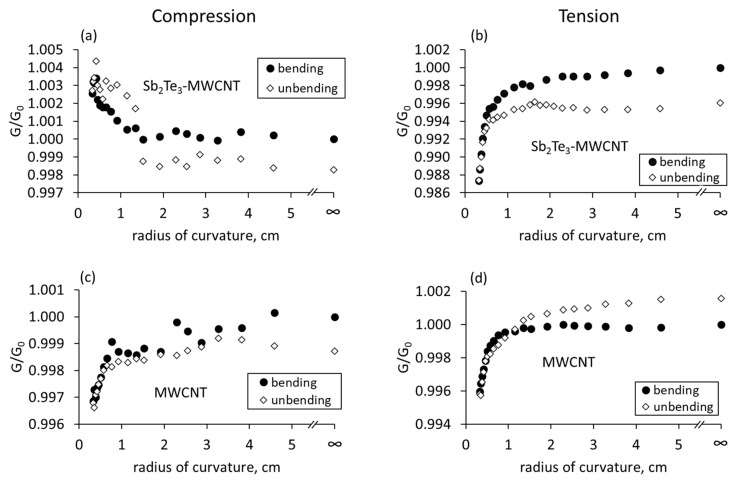
Relative changes in the electrical conductance of free standing Sb_2_Te_3_-MWCNT heterostructures and bare MWCNT networks deposited on polyimide surface vs. its radius of curvature: (**a**,**c**) compressive stress; (**b**,**d**) tensile stress. G is the conductance of the bent flexible film, G_0_ is the conductance of the not bent flexible film.

During the bending of free standing Sb_2_Te_3_-MWCNT heterostructures deposited on polyimide surface in the compression mode (Figure 4a), the electrical conductance starts to increase in the bending radii region starting from 15 mm and down to 3 mm.

The relative conductance changes when the sample is bent down to a 3 mm radius is ~0.5% in comparison to the unbent film. The opposite effect in the conductance change is observed when the side of the film with the highest Sb_2_Te_3_ concentration is subjected to bending in the tension mode. The conductance when bending down to a 3 mm radius decreases by 1–1.2% compared to the unbend sample (Figure 4b). In both bending modes when unbending the sample to the initial position, the conductance changes similarly to the forward bending the sample, and the conductance of the straightened sample is close to the initial values (Figure 4a,b). Presumably, the tendency of the conductance increase under compression and its decrease under tension during the bending is related to the uneven distribution of Sb_2_Te_3_ nanostructures in the sample, and to the dominating role of the Sb_2_Te_3_ layer on the changes in total conductance of the sample.

In the case of bare MWCNT networks with the uniform distribution of the MWCNTs, in both compression and tension bending modes the identical conductance decrease by 0.4–0.6% was observed when the sample is bent down to a 3 mm radius (Figure 4c,d). After the samples are straightened after bending, the electrical conductance of the MWCNT networks returns the initial conductance values of not bent sample with the maximal resistance deviations from the initial values of ~0.1% and ~0.2% for compressive and tensile stress respectively (Figure 4c,d).

**Figure 5:**
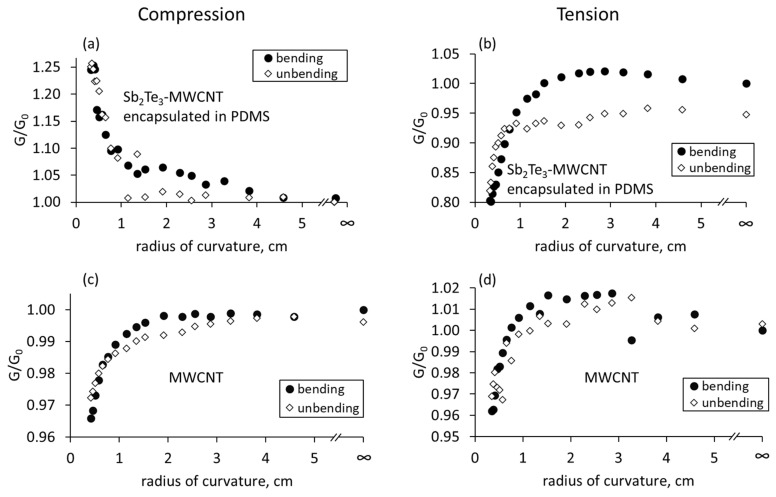
Relative changes in conductance of Sb_2_Te_3_-MWCNT heterostructures and bare MWCNT networks encapsulated in PDMS vs. its radius of curvature, while the side with higher Sb_2_Te_3_ concentration is subjected to compression (**a**,**c**) or tension (**b**,**d**). G is the conductance of the bent flexible film, G_0_ is the conductance of the not bent flexible film.

Encapsulation of Sb_2_Te_3_-MWCNT heterostructures in PDMS does not fundamentally affect the tendency of the conductance changes in the case of compression and tension (Figure 5a,b).

However, for the encapsulated heterostructures, the changes in the electrical conductance in compressive and tensile modes are by almost 40 times higher compared to the free-standing heterostructures, and the difference reaches approximately 20–25% of the initial conductance value (Figure 5a,b). Such differences can be arising both the polymer impact as well as the fact that the subjected to bending film with the encapsulated heterostructures is ~25 times thicker than the film with the free-standing heterostructures, and this can cause larger variations in the distances between the nanostructured components, resulting in larger changes in the electrical conductance. For the encapsulated bare MWCNT networks, the decrease in the electrical conductance by ~4% was observed for both compressive and tensile stress (Figure 5c,d). The return of the conductance close to the initial values when the sample is unbent after being subjected to either compressive or tensile stress (Figure 5a,b) may mean that the sample does not collapse and the distances between the nanoparticles and nanotubes return to the initial condition that the sample had before the bending. The reversible changes in the conductance may mean that the polymer between the nanostructures was compressed elastically.

**Figure 6:**
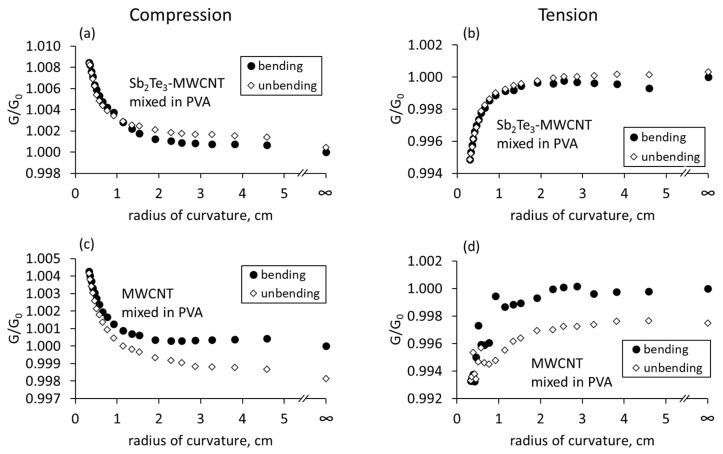
Relative changes in the electrical conductance of Sb_2_Te_3_-MWCNT heterostructures and bare MWCNT networks mixed in the PVA vs. its radius of curvature: (**a**,**c**) compressive stress; (**b**,**d**) tensile stress. G is the conductance of the bent flexible film, G_0_ is the conductance of the not bent flexible film.

For the films consisting of Sb_2_Te_3_-MWCNT heterostructures mixed in PVA, the tendencies of the conductance changes in compressive and tensile modes were similar changes observed for the free standing and encapsulated heterostructures (Figure 6a,b).

The conductance of these samples increased by 0.6–0.8% when subjected to compression (Figure 6a) and decreased by 0.4–0.5% when subjected to tension (Figure 6b) during the bending. However, in contrast with the free-standing and encapsulated samples, the behavior of bare MWCNTs-based samples prepared by mixing bare MWCNTs with PVA, was similar to the Sb_2_Te_3_-MWCNT heterostructures based samples, showing increase in the electrical conductance by ~0.4–0.6% when subjected to compression (Figure 6c) and its decrease by the same values when subjected to tension (Figure 6d). Presumably, such behavior of Sb_2_Te_3_-MWCNT heterostructures and bare MWCNT based samples mixed in PVA are related to their gradient distribution of the PVA matrix. Straightening of the thin films prepared by mixing method resulted in return of the electrical conductance to the initial values, indicating that the processes occurring in these films during the bending are reversible.

## 4. Discussion

**Figure 7:**
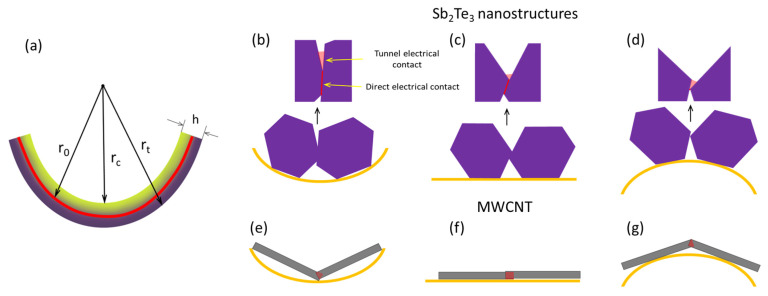
(**a**) a schematic of thin film bending; (**b**–**g**) a schematic representation of electrical contacts established between the Sb_2_Te_3_ nanostructures and MWCNTs in compression (**b**,**e**); not-bent (**c**,**f**), and tension (**d**,**g**) modes.

The experimental results can be interpreted using a thin film bending scheme (Figure 7a). Following this scheme, the side of the film, subjected to tension during the bending, will be stretched relative to the middle line of the thin film, representing the length of not-bent thin film, due to r_t_ > r_0_, causing separation of the components of the heterostructures. In contrast, the side of the film, subjected to compression stress, will be squeezed due to r_c_ < r_0,_ which will result in convergence of the components of the heterostructures. The ratios of the lengths of stretched or squeezed layers of the thin film relative to its middle line depend on the thickness of the thin film and can be calculated by simple formulas L_t_/L_0_ = (r_0_ + h/2)/r_0_ and L_c_/L_0_ = (r_0_ − h/2)/r_0_, where L_t_, L_c_ and L_o_ are respectively lengths of the thin film sides subjected to tension, compression, and the middle line, and h is the total thickness of the sample.

The conductance in the sample is determined by the number and type of electrical contacts established between the components of the heterostructures. The components of the heterostructured network may be in direct and/or tunnel electrical contact with each other (Figure 7c,f), both contributing to the total conductance of the sample.

The schematic shows only simplified contacts between two Sb_2_Te_3_ nanoparticles (Figure 7b–d) and between two MWCNTs (Figure 7e,f), and their changes for compressive and tension bending modes figures respectively. It is presumed in these modes, that the direct electrical contacts between the Sb_2_Te_3_ nanostructures, as well as between two MWCNTs are ohmic, however, the formation of Schottky barriers between the Sb_2_Te_3_ and MWCNT cannot be excluded. Subjection of the thin film to the compressive stress during its bending will result in convergence of the network components (MWCNTs, Sb_2_Te_3_ nanostructures), leading to the increase of the active area of previously established direct and tunnel electrical contacts between the nanostructures (Figure 7b), as well as to the establishment of a few new electrical contacts. The opposite process—distancing of the network components, resulting in a disruption or decrease of the active area of previously established between the nanostructures direct and tunnel electrical contacts—will occur when the film is subjected to the tensile stress during the bending (Figure 7d).

At low relative changes in the lengths of bent and not bent samples, it can be assumed that the increase or decrease of the active area of an electrical contact is proportional to the sample length change, and thus, the changes in the electrical conductance will be directly proportional to the changes in the active contact area. The changes in the tunnel current densities J when the sample is bent can be estimated using analytical equation for the electric tunnel effect between similar electrodes separated by a thin insulator film for rectangular potential barrier J = (6.2·10^10^/s^2^)(φ_0_ − V/2) exp (−1.025 s (φ_0_ − V/2)^1/2^) − (φ_0_ + V/2) exp (1.025 s (φ_0_ + V/2)^1/2^) [21], where J is the tunnel current density in A/cm^2^, s is the insulating layer thickness in Å, φ_0_ is the height of rectangular barrier in volts, and V is the voltage across the film. In the calculations, it was assumed that the height of the tunnel barrier is 4.45 eV [22] and 5 eV [23] for Sb_2_Te_3_ and MWCNT respectively, the initial barrier width of not bent sample is 1 nm, and the changes in the width of the potential barrier depended on the thickness of the film. The estimations of changes in linear dimensions of the thin films during the bending and related changes in the active areas of direct and tunnel contacts are summarized in Table 1. It can be seen from the Table 1 that the larger total thin film thickness is related to the larger changes the tunnel currents densities, as well as to the larger changes in the active area of the direct electrical contacts, and consequently, the larger changes in direct electrical current.

In what follows, the experimentally observed changes in the conduction of the Sb_2_Te_3_-MWCNTs heterostructures and bare MWCNTs based thin films are interpreted using the above models.

As is seen from the estimations shown in Table 1, the expected changes in the active electrical contact areas for the free-standing MWCNTs is the increase by ~0.3% (direct contact area) and 6.9% (tunnel current density) when the MWCNT network is subjected to compressive stress, and decrease by the same values (0.3% direct contact area, 7.5% tunnel current density) when the MWCNT network is subjected to tensile stress during the bending. Thus, the expected behavior of the free-standing MWCNT network would be a slight increase of the conduction in compression mode and decrease of the conduction in the tension mode. However, the experimental observations showed the decrease in the conductance of free-standing bare MWCNT networks by ~0.4–0.6% for both compression (Figure 4c) and tension (Figure 4d) bending modes. In the case of bare long MWCNTs, the direct electrical contacts may be dominating, and the contribution of the tunnel contacts to the total changes in conductance may be insignificant. In both compression and tension bending modes, long bare MWCNTs may slide relatively each other. Re-organizing (sliding) of the MWCNTs one relative to the other during compression (Figure 7e) or tension (Figure 7g) equally results in the decrease of the active area of direct electrical contacts between the CNTs and consequently, in the decrease of electrical conductance.

For the free-standing Sb_2_Te_3_-MWCNT heterostructures, the observed increase by 0.5% in the electrical conductance when subjected to compression (Figure 4a) and its decrease by 1–1.2% when Sb_2_Te_3_-MWCNT heterostructures were subjected to tension (Figure 4b). Such behavior is in agreement with the tendencies showed by the estimations (Table 1), and most likely is related to the distribution of Sb_2_Te_3_ in the films based on Sb_2_Te_3_-MWCNT heterostructures. In the Sb_2_Te_3_-MWCNT heterostructures, even though the conductance of the Sb_2_Te_3_ nanostructures itself is lower in comparison to MWCNTs, the larger impact of Sb_2_Te_3_ nanostructures on the conductance changes upon bending may be related to the smaller number of nanotubes compared to the number of Sb_2_Te_3_. Thus, the dominating contribution to the total conductance will be the electrical current through the Sb_2_Te_3_ nanostructures, with the higher contribution of the changes in tunnel contacts upon bending. Consequently, compression of the Sb_2_Te_3_ layer results in the formation of an additional number of new electrical contacts between the Sb_2_Te_3_ nanostructures and between the Sb_2_Te_3_ and CNTs, as well as in the increase of the active areas of direct and tunnel electrical contacts (Figure 7b, Table 1) and consequent increase of conductance. When subjected to tension, distraction of the Sb_2_Te_3_ nanostructures will result in disruption of a number of established electrical contacts, as well as in decrease of the active contact area of the electrical contacts (Figure 7d, Table 1), leading to the decrease in conductance. A slightly higher decrease in conductance when subjected to tension in comparison with its increase when subjected to compression (1–1.2% vs. 0.5%) may be explained by the contribution to the conductance decrease of the MWCNTs in the layers of Sb_2_Te_3_-MWCNT heterostructures, where MWCNTs are dominating, in both compression and tension modes, as it was observed for in the case of uniform distribution of the MWCNTs in the thin film.

For the encapsulated Sb_2_Te_3_-MWCNT heterostructures, the significant (~20–25%) increase in the electrical conductance of the encapsulated Sb_2_Te_3_-MWCNT samples when subjected to compression (Figure 5a) and its decrease by ~15–20% when subjected to tension (Figure 5b) may be explained by the impact of large total thickness of the film (~500 μm, Table 1), leading to the larger changes of the direct electrical contact area and consequently, in larger changes in direct (~7.7%) and tunnel (~−83.5% and +507% for tension and compression respectively) currents between the components of the heterostructures (Table 1). These tendencies in the conductance changes of the encapsulated Sb_2_Te_3_-MWCNT heterostructures, as well as the experimentally observed decrease in the conductance by ~4% of the encapsulated bare MWCNT networks when subjected to compression and tension during the bending (Figure 5c,d) allows presumption that the mechanisms of the conductance changes in the encapsulated samples are similar to ones in the free-standing samples, where conduction changes in the Sb_2_Te_3_-MWCNT heterostructures are governed by the processes occurring in the dense Sb_2_Te_3_ layer. However, despite the contribution of the decrease in the conductance of the MWCNTs in the lower layers of Sb_2_Te_3_-MWCNT heterostructures in both compressive and tension modes, the absolute values of the increase in the conduction, when the encapsulated Sb_2_Te_3_-MWCNT-based sample is subjected to compressive stress, is by ~5% higher than the absolute values of its decrease when subjected to tension (Figure 5a,b). This effect may be due to the significant increase in the tunnel current density in compression mode of the sample (~507%), which is ~6 times higher in comparison with the estimated tunnel current decrease in the tension mode (~83.5%, Table 1). In addition, the impact of PDMS on the interfaces between Sb_2_Te_3_ nanostructures in the Sb_2_Te_3_-MWCNT networks should be considered. Encapsulation is known to decrease the film overall conductance [7], possibly as a result of polymer penetration between the nanostructures. As the PDMS polymer is elastic, it may also change the distance between the nanostructures, as well as the height of the potential barriers between the nanostructures in case of tunnel electrical contacts. When the layer with the highest concentration of Sb_2_Te_3_ is compressed, it is possible that the polymer is compressed or partially extruded from the interface between the nanostructures, resulting in the reduction of the height of the potential barrier between the nanostructures, as well as the increase of direct electrical contact area between the nanostructures upon further compression.

In the samples, prepared by mixing the Sb_2_Te_3_-MWCNT heterostructures and bare MWCNTs in PVA, the increase by 0.6–0.8% and decrease by 0.4–0.5% in the electrical conductance when subjected to compression and tension respectively (Figure 6) is in agreement with the estimated for these samples changes: ~0.5% increase (when subjected to compression) or decrease (when subjected to tension, accompanied by the increase or decrease in the tunnel current by ~10.5–12% depending on the bending mode (Table 1). It should be noted that in contrast with the free-standing and encapsulated samples, where the active material is located at one side of the film, and consequently, is subjected to compression or tension during the bending, in the mixed samples the active material is present throughout the film. That means that during the bending of these films, while one side of the film is subjected to the compression, the opposite side of the film is subjected to tension, and the processes, occurring at both sides, contribute to the changes of total conductance of the film. During the mixing process of Sb_2_Te_3_-MWCNT heterostructures and bare MWCNTs with PVA, the components of the Sb_2_Te_3_-MWCNT heterostructures and MWCNTs are preserved in the PVA matrix apart from each other, forming direct and tunnel contacts. Gradient distribution and restriction of motion (sliding) of the MWCNTs in the PVA results in higher contribution of tunnel currents to the total conduction, and consequently, in bending behavior similar to the samples based on Sb_2_Te_3_-MWCNT heterostructures. During the bending, the impact of the sample side with higher concentration of nanostructures and MWCNTs is higher due to the higher number of newly formed, disrupted, or impacted contacts between them, which results in the increase of the electrical conductance when the sample is subjected to compression, and its decrease when the sample is subjected to tension.

## 5. Conclusions

In this work, bare p-type MWCNT networks and Sb_2_Te_3_-MWCNT heterostructures, fabricated by direct deposition of Sb_2_Te_3_ on MWCNT networks, were used for the preparation of three types of flexible films: free-standing networks on flexible polyimide substrates, encapsulated in PDMS, and mixed in PVA. Prepared films were subsequently subjected to compressive or tensile stress during the bending down to the radius of 3 mm, and the changes in the electrical conductance of the films in respect to the distribution gradient of the heterostructures or MWCNTs in these films was studied for the first time. It was found that the magnitude of the conductance changes was increasing with the increase of the total thickness of the film. All Sb_2_Te_3_-MWCNT heterostructures-based samples showed similar tendencies of the changes of the electrical response to bending, which was the increase of the electrical conductance under compressive stress and its decrease under tensile stress. Such tendencies most likely are related to the formation of new electrical contacts and/or convergence of the existing contacts between the Sb_2_Te_3_ nanostructures, MWCNTs, as well as between the Sb_2_Te_3_ and MWCNTs under compression stress, resulting in the increase of direct and tunnel contact areas, and to the disruption and/or distancing of previously established electrical contacts between these components under tensile stress, resulting in decrease of the direct electrical contact areas, transition of these contacts from direct to tunnel mode or disappearance of the contacts. Dominating contribution of Sb_2_Te_3_ nanostructures to the changes of electrical conductance under compressive stress of the Sb_2_Te_3_-MWCNT heterostructures-based films was proved by the data, obtained for the bare MWCNT-based films, were similar to the Sb_2_Te_3_-MWCNT heterostructures-based films behavior was not observed. Most of the bare MWCNTs-based samples showed a decrease in electrical conductance for both compressive and tensile stress. This effect is explained by the dominance of the direct electrical contacts in the MWCNT networks and equal decrease of the active contact area within the MWCNT network in both bending modes.

However, for the encapsulated Sb_2_Te_3_-MWCNT heterostructures, the changes in the conductance reached 20–25% from the initial conductance value. While the mechanism of the increase and decrease of the electrical conductance of the encapsulated samples was similar to the described above for the free-standing networks, significantly larger by the absolute values changes in the conductance can be explained by larger changes in the distances between the components of heterostructures during the bending due to the larger thickness of the film.

For the free-standing Sb_2_Te_3_-MWCNT heterostructures and MWCNT networks, as well as for the composites prepared by mixing them with PVA, the changes in the conductance remained within ~1% from the initial conductance value of not-bent sample. After unbending (straightening), the conductance values of all types of studied flexible films returned to the initial values with deviations not exceeding 0.5%. This proves stability of the investigated films and their suitability for the applications in flexible devices, for example, wearable electronics or low-power thermoelectrical generators. Expressed reversible changes in the electrical conductance of the encapsulated Sb_2_Te_3_-MWCNT heterostructures in response to bending indicated their usability for the applications on curved surfaces, as well as makes these films perspective for applications as strain-stress sensors. In addition, changes in electrical conductance when subjecting samples to compressive or tensile stress can be used to control the uniformity of distribution of components in materials composed of various heterostructures, as well as for the control of distribution of fillers in polymer-based composites.

## Figures and Tables

**Table 1:** Estimated changes in the length of sides (L), areas of direct electrical contacts (S), and tunnel current densities (J) of the polymer-based film subjected to tensile (L_t_; S_t_; J_t_) or compressive (L_c_; S_c_; J_c_) stress relative to the length of the middle line (L_0_), electrical contact area of not-bent sample (S_0_) and tunnel current density in not-bent sample (J_0_) of the film during the thin film bending down to the r_c_ = 3 mm.

Sample	h, μm	L_t_/L_0_	L_c_/L_0_	S_t,_/S_0_, %	S_c,_/S_0_, %	J_t_/J_0,_ %	J_c_/J_0,_ %
Free-standing on a flexible substrate Sb_2_Te_3_-MWCNT heterostructures	~20	~1.0033	~0.9967	−0.33	+0.33	−6.5	+7
Free-standing on a flexible substrate MWCNT network	~19	~1.0031	~0.9968	−0.31	+0.31	−6.9	+7.5
Sb_2_Te_3_-MWCNT heterostructures encapsulated in PDMS	~500	~1.0769	~0.9321	−7.69	+7.69	−83.5	+507
MWCNT networks encapsulated in PDMS	~499	~1.0755	~0.9245	−7.55	+7.55	−85	+574
Sb_2_Te_3_-MWCNT heterostructures mixed in PVA	~30	~1.005	~0.995	−0.5	+0.5	−10.5	+12
MWCNTs mixed in PVA						−11	+12.5

## Data Availability

The raw/processed data required to reproduce these findings cannot be shared at this time as the data also forms part of an ongoing study.
